# Lord of the fruit flies: an interview with Hugo Bellen

**DOI:** 10.1242/dmm.049500

**Published:** 2022-03-18

**Authors:** Hugo J. Bellen

**Affiliations:** Departments of Molecular and Human Genetics and Neuroscience, Duncan Neurological Research Institute, Baylor College of Medicine, 1250 Moursund, Houston, TX 77030, USA

## Abstract

During his remarkable career, Professor Hugo Bellen has innovated *Drosophila* genetics and forged a community driven toward diagnosis and treatment of rare diseases. He has advanced our understanding of nervous system development and neurodegeneration by exploring mechanisms and genetics through the latticed eyes of the common fruit fly. His lab, along with the labs of Shinya Yamamoto and Michael Wangler at Baylor College of Medicine and the Jan and Dan Duncan Neurological Research Institute of Texas Children's Hospital in Houston, also function as the *Drosophila* Core of the Model Organisms Screening Center (MOSC) of the Undiagnosed Diseases Network (UDN) and the Center for Precision Medicine Models. In this capacity, they facilitate the diagnosis of (ultra)rare human diseases and contribute to the development of treatments for these patients. Hugo is also the head of the *Drosophila* Gene Disruption Project supported by the National Institutes of Health (NIH) Office of Research Infrastructure Programs, and his lab channels substantial resources to the development of novel and sophisticated tools and technology that are then shared openly with the community via the Bloomington *Drosophila* Stock Center and the *Drosophila* Genomics Resource Center to propel research across the globe. Hugo has received an array of awards for his contributions to science and medicine, and he continues to be one of the most prominent figures in translational model organism research. In this interview, he discusses how his career progressed towards *Drosophila* genetics and highlights the accomplishments and challenges faced by the model organism community.


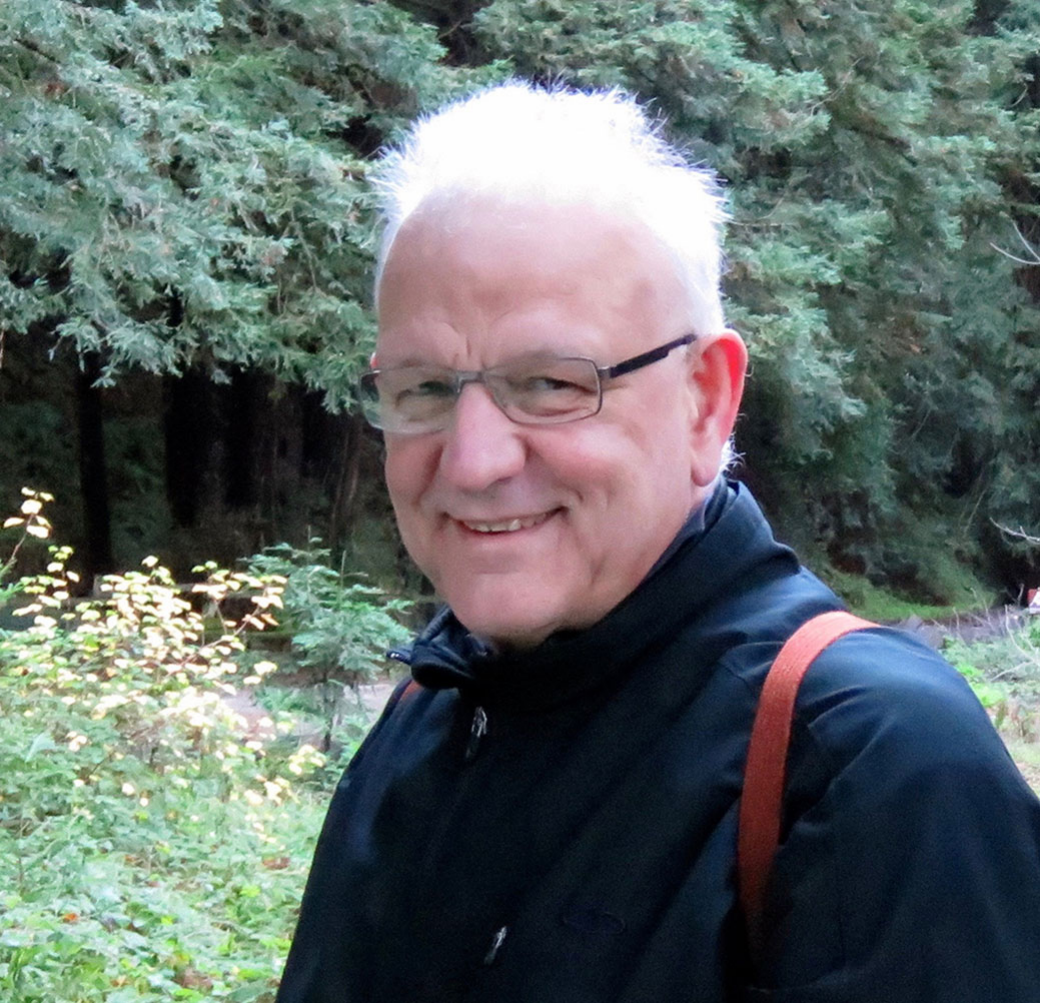


**What influenced your decision to do a PhD in genetics and how do you think your initial veterinary training benefits your research?**

I had a convoluted start. I first obtained a 5-year degree in engineering and economics at the University of Brussels Solvay School. I did an internship at Levi Strauss, but I was disappointed and decided that I needed to do something else. I was offered a different job as a researcher in mathematical economics at the University of Antwerp and worked on the weekends, as a hobby, in my friend's veterinary practice. After a year of this, I decided to go to vet school while still doing research on social welfare in Belgium. I really enjoyed veterinary medicine, and, in my third year of study, I had an outstanding genetics teacher, Jules Leroy, who sparked my interested in genetics. I immediately started thinking about doing research in a genetics lab. So, I joined an animal genetics research lab at the University of Ghent and worked on polymorphisms in rabbits, just to learn the basics. I finished my degree in veterinary medicine and applied for a PhD in genetics at the University of California, Davis. My plan was to work on a common disease in pigs, called malignant hyperthermia. This disease is prevalent in Belgian pigs and sometimes creates severe stress and death when the animals are transported. When I started talking to veterinarians about cloning the causative gene of this disease, their response was, “We don't even know which chromosome this gene is on – you're a bit ahead of the curve”. However, a *Drosophila* biologist who later became my mentor, John Kiger, said “You can do similar research in fruit flies”. So, I started my PhD working with fruit flies, and, once I started working with flies, I never looked back.

“Once you start working with flies, you get addicted. In flies, we can use sophisticated, classical genetics and generate transgenic animals to devise elegant strategies that address fundamental questions.”

**Why did you choose *Drosophila* to be the key model organism in your research?**

Once you start working with flies, you get addicted. In flies, we can use sophisticated, classical genetics and generate transgenic animals to devise elegant strategies that address fundamental questions. I know many other scientists who work on other model organisms and who envy what we can do with fruit flies. The approaches are often fast and inexpensive, and when you can manipulate an organism quickly and precisely, you can answer a lot of probing and important biological questions. This makes *Drosophila melanogaster* an outstanding model organism. When we're involved in the diagnosis of patients, the physicians now often prefer research using *Drosophila* because it quickly provides answers to their most pressing question: what is the impact of human variants on gene function?

“We were therefore able to identify the cause of this disease from one patient, *N* of 1, which is very difficult to establish in human genetics.”

**Your work with the UDN must be very rewarding. What inspired you to get involved with this?**

It started about 14 years ago, when we developed a screen in *Drosophila* for genetic mutations that are required for neuronal development and maintenance (Yamamoto et al., 2014). We screened for essential genes, the loss of which in the thorax leads to mechanosensory bristle loss and in the eye leads to progressive loss of vision. We identified and mapped over 600 mutations and identified 165 genes that fit these criteria, 93% of which were conserved between flies and humans. We then explored the Online Mendelian Inheritance in Man database (OMIM) to determine what fraction of the genes we identified in our screen are causing a human disease. At the time (∼2014), we found that 30% of the genes we identified had already been associated with one or more genetic diseases in humans. We then submitted our gene list to the Baylor-Hopkins Center for Mendelian Genomics (CMG) led by Jim Lupski and Richard Gibbs at Baylor College of Medicine. They responded very quickly to tell us that some of the genes identified in our screen had rare variants that segregated in some undiagnosed patients with rare diseases. This allowed us to establish the genetic diagnosis for different patients and families.

One of these patients carried rare variants in *ANKLE2* that caught our attention. This patient was born with an extreme microcephaly, so we looked at the brains of flies with a mutation in *Ankle2* and they also had very small brains. We then took the wild-type human gene, expressed it in the flies and fully rescued the brain size and survival of the flies. We also expressed the patient variants in the flies, which were not as efficient in rescuing the phenotype, so we knew the human disease was likely caused by these variants. We were therefore able to identify the cause of this disease from one patient, *N* of 1, which is very difficult to establish in human genetics. Around this time, Jim and Richard, asked me to speak about our collaborative efforts at the NIH on behalf of the CMG. Program officers and directors from the NIH approached me and asked, “How many genes could you identify in a year if you did this systematically?” I said, “A postdoc could do three per year, and if we had three or four postdocs, we could do 12 genes a year”. This would only be the beginning of a project, but it is often sufficient to establish a diagnosis. A little later came a request for applications (RFA) from the UDN of the NIH, as they wanted to set up a research center that performs genetic experiments based on flies and zebrafish to help with the diagnosis of variants in human genes that were not known to cause a disease, since this would be faster and cheaper than working with mice (Baldridge et al., 2021). We responded to this RFA together with Monte Westerfield and John Postlethwait at the University of Oregon, who both work with zebrafish. Our team was selected to become the first UDN MOSC, which was the first center to actively use model organisms for rare disease diagnosis.

We started working with the UDN in late 2015, and we published our first case already in 2017. The physicians and genetic counselors of the UDN would come to us with variants and genes they thought were the cause of a new rare disease, and we would quickly assess these in *Drosophila*. For diagnostic purposes it was very fulfilling, but I didn't realize until then how exciting it also was for parents and their families as they often have been on a diagnostic odyssey. After you publish one of these studies, very often the parents come to your office with their children, and you build up a relationship and become emotionally invested in the gene. Some of these parents establish patient organizations that contribute funding to the lab because they want to promote a better functional understanding of the disease-causing genes. They understand what we're doing with fruit flies and have even made recommendations about which novel drugs to test. We have tested a number of these drugs in flies and some really work well. Once we have compelling evidence that a compound works well to alleviate the phenotypes associated with a disease-causing gene, we work with physicians and apply for compassionate use at the U.S. Food and Drug Administration (FDA). Some of the drugs have had very positive effects on the patients. So, if you think about it, we started in 2015, and already we have diagnosed 35 new human diseases and have provided a better understanding of the molecular mechanisms for quite a few of them too. Seven children have or are being treated with drugs tested in fruit flies, some of which have really alleviated their symptoms. It's a step in the right direction.

“After you publish one of these studies, very often the parents come to your office with their children, and you build up a relationship and become emotionally invested in the gene.”

**Does any of your research in rare diseases shine light on more common diseases?**

The *ANKLE2* story had a very interesting sequel. A few years after publishing the initial study, I got a call from Nevan Krogan at the University of California, San Francisco. His lab had done an interactome study for proteins expressed by Zika virus. They expressed each Zika virus protein in human cells and looked for all the proteins that interact with the ten viral proteins. They only found a few human proteins out of hundreds of proteins identified that interact with the Zika proteins and were previously associated with microcephaly, a hallmark symptom of children infected in the womb during early pregnancy. One of these was ANKLE2. Their study identified a Zika protein, NS4A, that robustly binds to ANKLE2, but they had no idea if this interaction was related to the microcephaly associated with the Zika virus epidemic in South America. Nevin asked me whether we were still working on *Ankle2* and whether we could test its interaction with viral proteins in *Drosophila*. We said yes, and Nikki Link, a former postdoc in my lab, expressed the NS4A protein from the Zika virus in the fly, and their brains were even smaller than flies with partial loss of *Ankle2*. Then, she found that co-expressing the wild-type human ANKLE2 protein fully suppressed the small brain phenotype caused by NS4A in flies and characterized the pathway in which *Ankle2* plays a role in neuroblasts. These data indicate that the NS4A viral protein and the human ANKLE2 protein are key players in microcephaly caused by the Zika virus (Shah et al., 2018; Link et al., 2019).

When I started talking to physicians in Brazil about Zika, I heard that there were entire clinics of physicians just devoted to microcephaly caused by Zika. That is when I realized that, although most of our studies start by working on a rare disease, our work can also impact people suffering from more common diseases. This has become a research theme in the lab. When we find the mechanism of how specific variants in genes cause a rare disease, we often find that other genes that work in the same pathway have been identified in genome-wide association studies or other studies of common diseases. More specifically, we came to realize that neurological diseases that are very severe in early stages of life share pathways with late-onset diseases like Alzheimer’s disease, Parkinson's disease and multiple sclerosis, but the diseases in later life affect the pathway in a much more subtle way. That's really interesting because homozygous loss of a gene in these rare developmental disorders gives us insight about the heterozygous loss or gain of gene function that is much more subtle in some adult-onset diseases. Hence, several members of the lab are now working on common diseases including Alzheimer's disease, Parkinson's disease and multiple sclerosis, which all started from work investigating very rare diseases (Liu et al., 2017; Moulton et al., 2021).

**Does opening up your research to other, more common diseases have an impact on funding or interest from pharmaceutical companies?**

Large pharmaceutical companies are typically not interested in rare diseases. They're mainly interested in supporting research and drug development for common diseases. Rare diseases are not really on their radar. There are a few small biotech companies that are investing in rare diseases, but they typically do not support in-depth mechanistic research.

The parent organizations, on the other hand, are eager to have animal models of the rare diseases, and with some mouse models generated through their financial support, we're testing gene therapy. Guang Lin and Burak Tepe, two fellows in my lab, are involved in a preclinical gene therapy study in mice supported by the INADCure Foundation [parent foundation for Infantile Axonal Dystrophy (INAD)], and we have been trying to get support from companies, but the companies we have been in touch with so far aren't interested in the rare disease aspect. They are, however, interested in the fact that some of these rare diseases like INAD have links to Parkinson's disease or other more common diseases (Lin et al., 2018). If we can get drugs that work for these rare diseases, and they prove to be effective in fly models for Parkinson's disease, then they may become interested. But we're not there yet, so currently some parent organizations support us.

**It is amazing what these patient advocacy groups can do, and how they can bring together patients and families from all over the world**

After we publish a manuscript regarding a novel rare disease that we discovered in collaboration with clinicians, I get emails from parents who state that their child was undiagnosed, but that their physician analyzed their genomic sequence and found that the gene we just published is the cause of their disease. And if these parents are a bit proactive, they start a group on social media and identify additional patients affected by the same condition around the world. For some of the diseases, we've published the original study with as few as three patients, and now there are up to 300 people in these groups, demonstrating the power of social media and networking catalyzed by patients and their family members. You start with a really rare disease, and you think it's never going to be found in a patient again, but, for some of these diseases, there are numerous patients within a few years of the discovery. The support of these groups has been very valuable to pursue the study of some genes to identify disease mechanisms and potential drug targets.

**How important is it to be, not only at the forefront of developing novel technology and tools, but also then sharing this with other labs globally?**

I think that's another reason why the fruit fly field is competitive with other model organism research. I realized how important technology development was a long time ago, when I was a postdoc in Walter Gehring's lab and teamed up with Cahir O'Kane (Cambridge University) and Clive Wilson (Oxford University). Together, we embarked on generating tools to see how well we could manipulate *Drosophila* with transposable elements to help us determine gene expression and function (Bellen et al., 1989; Wilson et al., 1989).

Subsequently, I joined Baylor College of Medicine as an assistant professor. I was lucky to get funded by the Howard Hughes Medical Institute and immediately decided that, given that I did not have to devote a significant amount of my time to writing grant proposals, I should give back to the community. I decided to develop tools and reagents, and deposit them in the Bloomington *Drosophila* Stock Center to promote the field. In the beginning, I didn't get funding from the NIH to do this, but, around the year 2000, Gerry Rubin and Allan Spradling teamed up to help me secure funding and continue this project. Then, scientists, like Koen Venken, who were interested in developing technology, applied to my lab. This was exciting because I really wanted to continue to develop new tools. Together, we built up a team of people to develop new technology and resources, including many technicians as these projects are very large. Much of what we do is really driven by these new technologies, as they speed up the generation of reagents and increase the precision and depth of assessing gene function. This allows anyone using flies to study mechanism at a much, much greater pace. Many other model organism researchers want to develop similar technologies as they are the driving force to further our understanding of biology.

If someone develops a technology and it's used by few, it's a contribution, but if they can implement the technology to every gene, share the reagents via public stock centers like the Bloomington *Drosophila* Stock Center, and more than 1000 people use it, then they have made an impact. The *Drosophila* Gene Disruption Project, for example, is a big effort for my lab; there are 15 technicians, postdocs and a junior faculty member, Oguz Kanca, in my lab, who together with a team in Norbert Perrimon's lab at Harvard, develop new technology and resources (Kanca et al., 2019). We have also been working with Susan Celniker at Laurence Berkeley National Laboratory and Shinya Yamamoto at Baylor College of Medicine to generate a collection of transgenic *Drosophila* lines that allow expression of thousands of human proteins. Centralization like this has a huge advantage, because the protocol is standardized, there's a systematic production pipeline, and it's efficient, fast and reliable. There is an ‘economy of scale’ that allows us to create reagents much cheaper than if individual reagents were generated by individual labs.

As soon as new transgenic *Drosophila* lines are made, they are sent to Bloomington and we don't keep any for ourselves. Obviously, when genes from clinicians are submitted to us, we have the privilege to put those on a priority list. However, we generate about 500-600 *Drosophila* lines a year, and genes for the UDN only make up a small percentage of those. One key principle that I follow is that the technology and resources developed in my lab have to be simple to use and do not require sophisticated equipment. Technology that is complicated and costs a lot of money to implement is not what I'm looking for. I'm looking for strategies that most fly biologists can use readily.

**You wrote a Perspective article for our sister journal, Development, that looked at the challenges the model organism databases are facing due to NIH funding cuts (Bellen et al., 2021). Could you explain why this is so important for model organism researchers, geneticists and clinicians?**

I'll start by explaining how the UDN generally works so that you understand why model organism databases are not only important for basic research but also for clinical research and practice. In the UDN, patients accepted into the study are clinically evaluated by one of 12 clinical sites around the country. These sites carry out extensive phenotyping of the patient, using state-of-the-art clinical resources and techniques. Next, Baylor Genetics sequences the genomes of the patient and relatives enrolled in the UDN. In more than 50% of the cases, they still don't know which gene is responsible for the patient's phenotype, but they will provide a list of genes and variants that they've identified based on what's known in the literature. The physicians then look at these candidates, and sometimes they can solve the case by identifying other patients with similar genotypes and phenotypes, or by performing functional studies in collaboration with a model organism researcher or cell biologist. If they can't solve it, they can submit it to the MOSC. These are often the most difficult cases, which are dubbed ‘medical mysteries’ in the media.

“Model organism researchers rely on these databases almost daily – the databases are the lifeline for their research.”

The first thing we, as MOSC researchers, need to do is to go to various databases. We need to mine as much data as we can about the candidate variant and gene, not only using human datasets but prior knowledge accumulated in model organisms. We have integrated many databases in a search tool, MARRVEL (Wang et al., 2017), in collaboration with Zhandong Liu, a bioinformatician and data scientist. It integrates numerous human genetics databases as well as model organism databases to try to gather as much information in the shortest possible amount of time. We developed MARRVEL because we previously spent a lot of time navigating numerous databases and learning how to extract useful information during the initial phase of the project. Once we run everything through MARRVEL and other specialized clinical databases, UDN physicians and model organism researchers with expertise in fly, worm and zebrafish genetics discuss the case based on all the available information. Through this discussion, we select the best actionable plan, including a decision on which model organism to use to help diagnose the case. If we didn't have all this detailed information from all the model organism databases, and from all the human databases, we would be nowhere. We couldn't even initiate the bench work. Model organism researchers rely on these databases almost daily – the databases are the lifeline for their research.

So, what happened many years ago was that the National Human Genome Research Institute (NHGRI) decided to support the model organism databases. This comes at a serious cost of around $25 million a year to support all the databases, including yeasts, worms, flies, zebrafish, *Xenopus*, rats and mice. However, more recently, the NHGRI became somewhat disappointed that all the information in the separate model organism databases was so difficult to access and mine for researchers who do not have the expertise in specific organisms. So, they supported the start of the Alliance of Genome Resources (AGR), which is an integrated database where all model organism databases deposit their data in a standardized way that allows cross-database searches by informaticians and geneticists. This is an integrated database curating information from different species, which is a good goal, and is precisely what we're trying to achieve with MARRVEL.

In order to provide support for AGR, NHGRI decided to decrease the support for each model organism database. The rationale was that integrating tools such as artificial intelligence (AI) should allow us to reduce operating costs. AI can certainly aid in some manuscript curation, but one needs experienced curators to extract information properly and link all the info. We need curators to look at these data and make logical connections between many datasets. It's too complicated to rely solely on AI, even with the best algorithms. AI may be able to take over some of the duties, but manual curation by experts is essential. Moreover, the information required for each species is highly specialized and often not relevant for other species, and individual model organism databases will remain essential. AGR may become a valuable resource for the community and will facilitate data mining, but without sustained support of individual model organism databases, which provide the primary data for AGR, AGR is also at risk of becoming inefficient.

I think we should invest more money in the model organism databases, rather than less. They only take up a fraction of a percent of what's invested in model organism research by the NIH, but these databases return many times more than what the NIH invests in them. They are absolutely essential resources, but there's a lack of appreciation since many people take the existence of databases for granted. Loss of support is going to lead to a duplication of effort, duplication of investment in resources, loss of information and unnecessary competition between investigators. There's no way we can mine the entire literature in a comprehensive way without the databases. It's just mind-boggling and upsetting that this is happening. I'm not alone in this fight and I think it's really important that we step up to the plate to make sure there is a remedy to this.

**Thank you for providing your insight on such an important topic. To end on a more light-hearted note, what do you usually do outside of work?**

I work almost every day, as I really enjoy what I'm doing, but my main hobby is fly fishing. Also, my spouse, Catherine Tasnier, and I love hiking, so once in a while we take a few weeks off to go hiking.
